# Monolithic ultrasound fingerprint sensor

**DOI:** 10.1038/micronano.2017.59

**Published:** 2017-11-20

**Authors:** Xiaoyue Jiang, Yipeng Lu, Hao-Yen Tang, Julius M. Tsai, Eldwin J. Ng, Michael J. Daneman, Bernhard E. Boser, David A. Horsley

**Affiliations:** 1 Department of Mechanical Engineering, University of California, Berkeley, CA 94720, USA; 2 Department of Mechanical and Aerospace Engineering, University of California, Davis, CA 95616, USA; 3 Department of Electrical Engineering and Computer Sciences, University of California, Berkeley, CA 94720, USA; 4 Invensense Inc., San Jose, CA 95110, USA

**Keywords:** piezoelectric micromachined ultrasonic transducer (PMUT), fingerprint sensors, ultrasound

## Abstract

This paper presents a 591×438-DPI ultrasonic fingerprint sensor. The sensor is based on a piezoelectric micromachined ultrasonic transducer (PMUT) array that is bonded at wafer-level to complementary metal oxide semiconductor (CMOS) signal processing electronics to produce a pulse-echo ultrasonic imager on a chip. To meet the 500-DPI standard for consumer fingerprint sensors, the PMUT pitch was reduced by approximately a factor of two relative to an earlier design. We conducted a systematic design study of the individual PMUT and array to achieve this scaling while maintaining a high fill-factor. The resulting 110×56-PMUT array, composed of 30×43-μm^2^ rectangular PMUTs, achieved a 51.7% fill-factor, three times greater than that of the previous design. Together with the custom CMOS ASIC, the sensor achieves 2 mV kPa^−1^ sensitivity, 15 kPa pressure output, 75 μm lateral resolution, and 150 μm axial resolution in a 4.6 mm×3.2 mm image. To the best of our knowledge, we have demonstrated the first MEMS ultrasonic fingerprint sensor capable of imaging epidermis and sub-surface layer fingerprints.

## Introduction

Fingerprint sensors capture an electronic image of a human fingerprint through various physical mechanisms, including optical, capacitive, pressure and acoustic mechanisms. Capacitive fingerprint sensors are the standard for identity authentication in numerous applications because of their performance and low cost; the latter is due to the fact that these sensors can be manufactured in a standard integrated circuit manufacturing process. Ultrasonic fingerprint sensors have many advantages over capacitive sensors, including being insensitive to contamination and moisture on the finger. In addition, ultrasonic waves used in pulse-echo imaging can penetrate the finger’s epidermis, collecting images of sub-surface features. However, ultrasonic fingerprint sensors previously provided a low resolution or were too difficult to manufacture. With the rapid development of microelectromechanical systems (MEMS) technology, micromachined ultrasonic transducers (MUTs) based on capacitive (CMUT) and piezoelectric (PMUT) transduction have been demonstrated with significantly reduced device sizes for high-resolution applications, low power consumption and better acoustic impedance matching to the medium^1,2^. In general, CMUTs suffer from limited vertical deformation, nonlinear drive effects and high DC bias voltages, but they have high electromechanical coupling factors^3^. With improvements in piezoelectric materials technology, PMUTs are beginning to pose an alternative to CMUTs. The most widely available thin-film piezoelectric materials for PMUTs are lead zirconium titanate (PZT) and aluminum nitride (AlN). PZT has better piezoelectric properties, but it is not complementary metal oxide semiconductor (CMOS)–compatible and may suffer from ageing and other material property changes over time^4^. By contrast, AlN is lead-free, has low-temperature (<400 °C) deposition and has demonstrated stable material properties in the mass production of AlN bulk acoustic-wave (BAW) filters. However, AlN has lower piezoelectric coefficients than PZT. A monolithic ultrasonic fingerprint sensor based on an 8×24 AlN piezoelectric micromachined ultrasonic transducer (PMUT) array with 254-DPI pitch has recently been demonstrated^5,6^. However, the standard for fingerprint sensors in consumer electronics is 500 DPI, requiring a dramatic reduction in the PMUT size to <50 μm, risking both low acoustic output and reduced fill-factor because the active acoustic area shrinks while the area needed for anchors and interconnect does not. This paper presents a systematic design study of the individual AlN PMUT and array parameters to resolve these issues.

Sensors based on dense 2D MUT arrays require integration with CMOS to enable signal multiplexing to thousands of MUTs in the array. CMUTs have been integrated with CMOS using through-silicon vias (TSVs) and solder-ball flip-chip bonding^7^. However, both TSVs and flip-chip bonding are relatively expensive processes. More importantly, the minimum solder ball diameter is approximately 80 μm (Ref. 8), making them unsuitable for electrical interconnect to individual MUTs in a 500 DPI array, where the pitch between MUTs is 50 μm or less. Here, a MEMS-CMOS eutectic wafer-bonding process used for high-volume manufacturing of inertial sensors was adapted to produce PMUT arrays, enabling each PMUT to be directly bonded to a dedicated CMOS receive amplifier to minimize electrical parasitics^9^. In an earlier fingerprint sensor designed with this technology, a 17% fill-factor PMUT array achieved 14 kPa peak-to-peak pressure output, 0.6 μV Pa^−1^ sensitivity and 200 μm image resolution^6^. In this study, a 51.7% fill-factor, 110×56 array of rectangular PMUTs is demonstrated, an increase of 140% pressure output per input voltage along with over 200% in sensitivity and image resolution.

## Materials and methods

**Figure 1:**
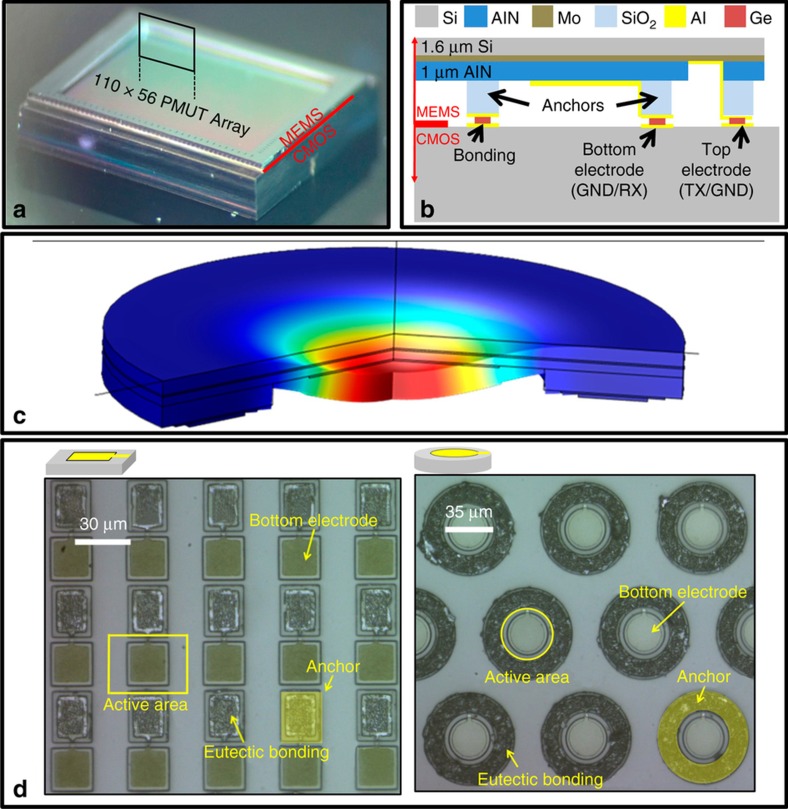
(**a**) Photograph of the sensor. The 110×56 piezoelectric micromachined ultrasonic transducer (PMUT) array is located in the 4.64 mm × 3.36 mm recessed region in the center of the die. (**b**) PMUT cross-section schematic. (**c**) Finite element method (FEM)–simulated mode shape of a circular PMUT with an applied electric field across the AlN piezoelectric thin film. (**d**) Optical images of the two PMUT arrays after debonding from the complementary metal oxide semiconductor (CMOS) wafer. In the rectangular design (left), a single Al-Ge anchor (highlighted in yellow) mechanically isolates PMUTs in adjacent rows, while PMUTs in adjacent columns are not mechanically isolated. The circular design (right) sacrifices fill-factor for more anchor area and increased spacing between the Al-Ge bonding rings.

A cross-section schematic of an individual PMUT is shown along with photographs of the 110×56 sensor chip in Figure 1a. Each PMUT is a piezoelectric unimorph composed of 1-μm-thick AlN sandwiched between 300 nm Al and 200 nm Mo electrodes on a single-crystal silicon layer with 1.6 μm nominal thickness (Figure 1b). Al-Ge eutectic bonds on SiO_2_ standoffs provide the mechanical anchor and electrical contact to the PMUT^9^. The deformation of a PMUT with an external electrical field applied to the AlN is shown in Figure 1c. The PMUTs are formed on an SOI MEMS wafer that is bonded to a CMOS wafer that provides the signal processing electronics, which includes the 24 V charge pump, high-voltage transmit amplifiers, low-voltage receive amplifiers and multiplexing circuitry. Details regarding the design of the signal processing circuitry are provided in Ref. 10. Following CMOS-MEMS wafer bonding, the PMUTs are released by a through-wafer DRIE etch that removes the MEMS handle wafer in a 4.6-mm × 3.2-mm region at the center of each 5.4 mm × 4.6-mm die. Two 110×56-element array designs, composed of 35-μm-diameter circular and 30-μm×43-μm rectangular PMUTs, were fabricated. The optical images in Figure 1d show the layout of the rectangular and circular PMUT arrays. Each of the 6160 PMUTs in the array has a dedicated receive (RX) amplifier that is connected to the Al bottom electrode during the receive phase. The 56 PMUTs in each column share a common Mo top electrode that is connected to a 24 V transmit amplifier (TX) during the transmit phase. To protect the RX amplifier from the high voltage signal, a TX/RX switch connects each bottom electrode to ground during the transmit phase.

### Individual PMUT design

**Figure 2:**
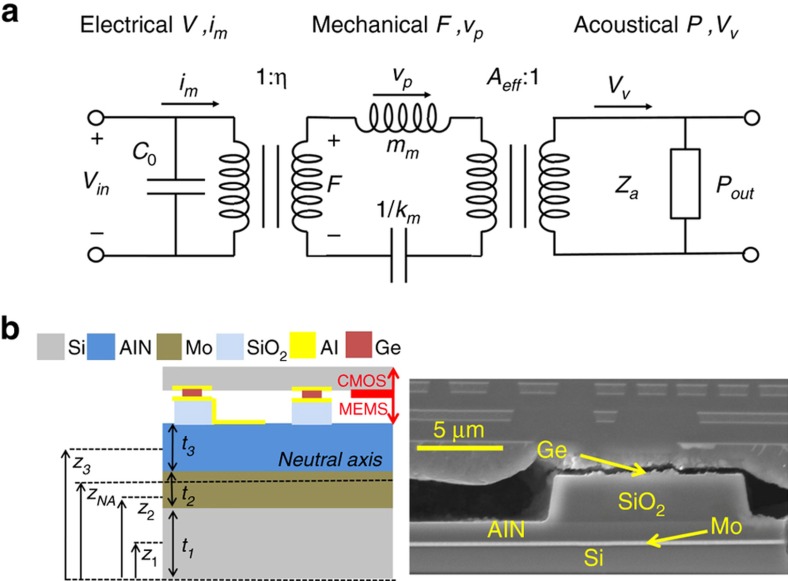
(**a**) Electrical–mechanical–acoustic model. Voltage *V* and current *i*_m_, force *F* and velocity at the center of the PMUT *v*_p_, and pressure *P* and volume velocity *V*_v_ are the variables for the electrical, mechanical, and acoustical domains, respectively. (**b**) Schematics (left) and scanning electron microscope (SEM) image (right) of a PMUT cross-section. The Ge bond in the SEM image was broken during sample preparation.

The electrical–mechanical–acoustic equivalent circuit model for the PMUT, shown in Figure 2a, is used to understand the resonant frequency in air and in fluid, peak velocity and peak pressure at the center of the PMUT^2,11^. In the mechanical domain, equivalent circuit parameters are derived to represent the mass *m*_m_ and stiffness *k*_m_ for a particular vibration mode. The mechanical parameters, force *F* and velocity *ν*_P_ (measured at the center of the PMUT), can be converted to the electrical domain using the electromechanical coupling constant *η*. The output force is converted to an output pressure by dividing by the effective surface area *A*_eff_, which is one-third of the total area for a circular PMUT. In the acoustic domain, the acoustic impedance *Z*_a_ relates the acoustic pressure *P* to the volume velocity *V*_ν_ as *P*=*Z*_a_*V*_ν_.

The parameters of the equivalent circuit model are derived as follows. A schematic and scanning electron microscope (SEM)cross-section of the PMUT is shown in Figure 2b. While the Al bottom electrode does not span the entire PMUT surface, this layer is thin and the Young’s modulus of Al is low relative to that of the other (Si, Mo, AlN) layers, so we neglect it in the following derivations. For a unimorph PMUT composed of three layers, the neutral axis for the PMUT laminate, shown in Figure 2b, can be calculated as^2^

(1)zNA=∑n=13tnznE′n∑n=13tnE′n

where *n* is the layer index (Si, Mo, AlN), ${E\prime }_{n}=E_{n}/\left(1-\upsilon_{n}^{2}\right)$ is the plate modulus, *E*_*n*_ and *υ*_*n*_ are the layer’s Young’s modulus and Poisson’s ratio, *z*_*n*_ is the distance between the layer’s middle plane to the bottom of the laminate and *t*_*n*_ is the layer thickness. The mass per unit area *μ* is^2^

(2)µ=∑n=13tnρn

where *ρ*_*n*_ is the density of the *n*th layer. Meanwhile, the flexural rigidity for a laminate is defined as^12^

(3)D=13∑n=13E'n(h¯n3−h¯n−13)

where ${\bar{h}}_{n}=h_{n}-z_{NA}$ is the distance between the top the *n*th layer to the neutral axis.

Assuming a circular plate vibrating in the first mode, the modal stiffness, modal mass and electromechanical coupling constant are given by

(4)km=64πD3a2

(5)mm=πa2µ5

(6)η=4πγ2(γ2−1)e31,fz¯p

where *a* is the radius of the PMUT, *γ* is the ratio of the Al bottom electrode radius to the PMUT radius, *e*_31,*f*_=1.08 C m^−2^ is the effective thin-film piezoelectric coefficient of AlN^13^ and ${\bar{z}}_{p}$ is the distance from the middle of the piezoelectric layer to the neutral axis. In this paper, we use the velocity at the center of the PMUT as the mechanical velocity variable because this value is measurable in experiments. In other works, the average velocity $\bar{\nu }=v_{P}/3$ is often chosen, a choice that yields $\overset{¯}{k_{m}}=9k_{m}$ and $\overset{¯}{m_{m}}=9m_{m}$, values that are consistent with Mason’s approach^14^. In the acoustic domain, the acoustic impedance *Z*_a_ for a clamped radiator can be found as^15^

(7)Za=ρcAeff(rr+jxr)

where *R*_*r*_ and *x*_*r*_ are the resistive and inductive acoustic terms, respectively. The imaginary part of the acoustic impedance behaves as a mass *m*_a_ added to the PMUT mass in the mechanical domain.

Circular PMUTs with dimensions from 35 to 70 μm were modeled, fabricated and characterized. For a unimorph PMUT with total thickness *t* and characteristic length *l* (diameter or side length), the resonant frequency of the fundamental flexural vibration mode in air is given by

(8)2πf0,air=kmmm∝tl2

**Figure 3:**
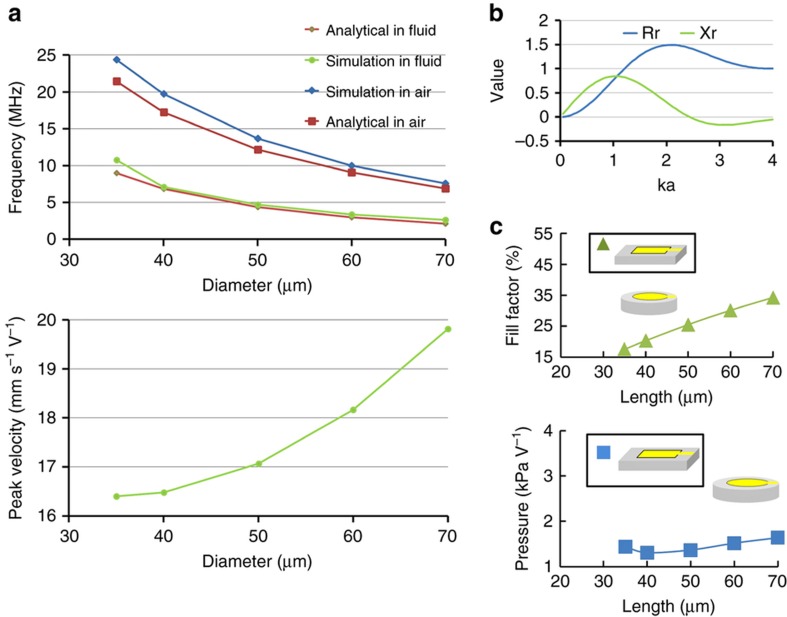
(**a**) Frequency response in air and fluid from the simulation and analytical solution (top). Peak velocity from the analytical solution (bottom). (**b**) The resistive *r*_r_ and inductive *x*_r_ terms in the acoustic radiation impedance plotted versus *ka*. (**c**) Fill-factor (top) and computed pressure output (bottom) versus PMUT size.

The resonant frequency of a PMUT immersed in fluid can be estimated from Horsley *et al*

(9)f0,fluid/f0,air≈mmmm+ma≈1/1+0.34ρfluidlµ

where *ρ*_fluid_ is the fluid’s density. The resonant frequencies predicted by Equation (8) and Equation (9) agree well with resonant frequencies from a finite element methodmodel of the PMUT in air and in fluid (COMSOL Multiphysics), as shown in Figure 3a. The results show that the first resonance frequency *f*_0_ of a PMUT immersed in a fluid scales as 1/*l*^2^, as predicted by the analytical models. Meanwhile, the displacement at the center of the PMUT *d*_*p*_ is given as^17^

(10)dp=Qds∝e31znBWDf0∝l2t4

where *Q* is the quality factor, *d*_s_ is the static displacement and *BW* is the 3 dB bandwidth of the PMUT. The velocity at the membrane center is

(11)vp=2πf0,fluiddp∝1/t3

Whereas Equation (11) suggests that the velocity should be independent of diameter, and the calculated velocity, shown in Figure 3a, shows that the velocity changes slightly with diameter, increasing by 20% with a 200% increase in diameter. Finally, the pressure output scales with the effective area, velocity and acoustic impedance are given as

(12)Pp=ZavPAeff

The resistive and inductive acoustic impedance terms are functions of the product of the wave number *k* and radius *a*, shown in Figure 3b. For an individual PMUT with 1-μm AlN and a 1.6-μm silicon device layer, when the diameter is doubled, the output pressure and peak velocity increase by <25%, while the resonant frequency in fluid decreases by 75%.

### Array design

On an individual basis, there is little difference between the circular and rectangular PMUTs when designing for output pressure. However, when individual PMUTs are arranged into an array, scaling the PMUT causes the active acoustic area to shrink while the area needed for anchors and interconnect does not. Defining the fill-factor *F* to quantify the active acoustic area and comparing designs that can achieve a 50-μm pitch, a 35-μm circular PMUT results in a 17.6% fill-factor, while a 30 μm×43 μm rectangular PMUT results in a 51.7% fill-factor, a factor of 3 better. A simple model for the ideal surface pressure generated by a surface oscillating with amplitude *d*_p_ at frequency f_0_ is given as^6^

(13)P=(2πf0dp)ZaAeffF

where *F* is the fill factor of the array. Figure 3c shows the best possible fill factor calculated for PMUTs with different sizes and the computed surface pressure based on simulated peak displacement. The calculated pressure output suggests that the rectangular PMUT array design can achieve twice the pressure output of the circular PMUT array.

**Figure 4:**
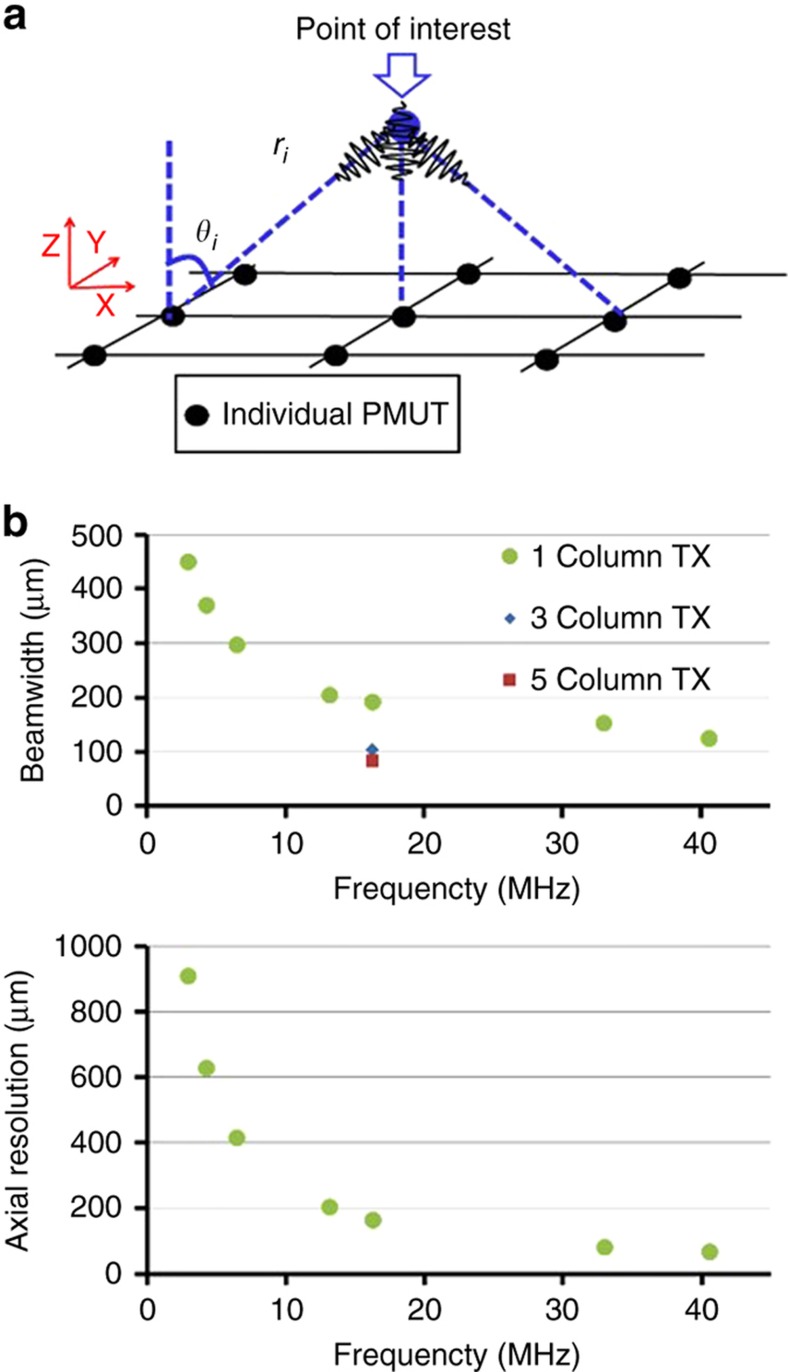
(**a**) Schematic of the superposition of the pulsed pressure outputs from the excited piezoelectric micromachined ultrasonic transducers (PMUTs) in the array. (**b**) Beam width (top) and axial resolution (bottom) versus the operating frequency of PMUT in fluid. Beam width calculations were performed when a single column is used to transmit, as well as when three columns and five columns are used.

For pulse-echo-based ultrasound imaging, lateral resolution depends on the beam width, while the axial resolution is determined by the product of the wavelength and number of cycles in the transmitted pulse^18^. The total pressure output at a point of interest is the superposition of the pressure output from all the PMUTs^19^,

(14)Ptot=∑Pavrika22De(θi)e−jkriφ(t)

(15)De(θi)=48J3(kasinθi)(kasinθi)3

where *P*^av^ is the average output pressure, *D*_e_(*θ*_i_) is the directivity, *φ*(*t*) is the normalized pulse signal and *θ*_i_ and *r*_i_ are the angle and radial distance between the PMUT and the point of interest, respectively. The schematic of the superposition of the pressure outputs from the PMUT array is shown in Figure 4a. In experiments, the PMUTs are covered by a 250 μm-thick layer of polydimethylsiloxane (PDMS) (Sylgard 184, Dow Corning, Midland, MI, USA), and the speed of sound of PDMS (*c*=1000 m s^−1^) is used to calculate the wave number in the model, *k*=2π*f*/*c*≈10^5^ m^−1^ at 16 MHz. The PMUTs in each column of the array share a common top electrode, which is excited with the transmit voltage. Because the column length in the vertical (*y*) axis is much greater than its width in the horizontal (*x*) axis (3.2 mm vs. 70 microns per column), the *x*-axis beam width is of most significance. Therefore, the expected beam width at the imaging plane (which is the surface of the PDMS layer), 250 μm above the transducer array, is calculated for a column of PMUTs operating in PDMS with the expected frequencies for seven PMUT diameters ranging from 25 to 70 μm, as shown in Figure 4b. As the frequency increases, the beam width decreases dramatically, leading to better lateral resolution. Beam-width calculations were performed for single column excitation, as well as for cases where three columns and five columns are transmitting together. As shown in Figure 4b, the increased aperture when transmitting with three columns and five columns without beamforming results in greater resolution. The calculated beam-width for this design at 16 MHz is 104 μm with three columns transmitting and 84 μm with five columns transmitting.

## Results

**Figure 5:**
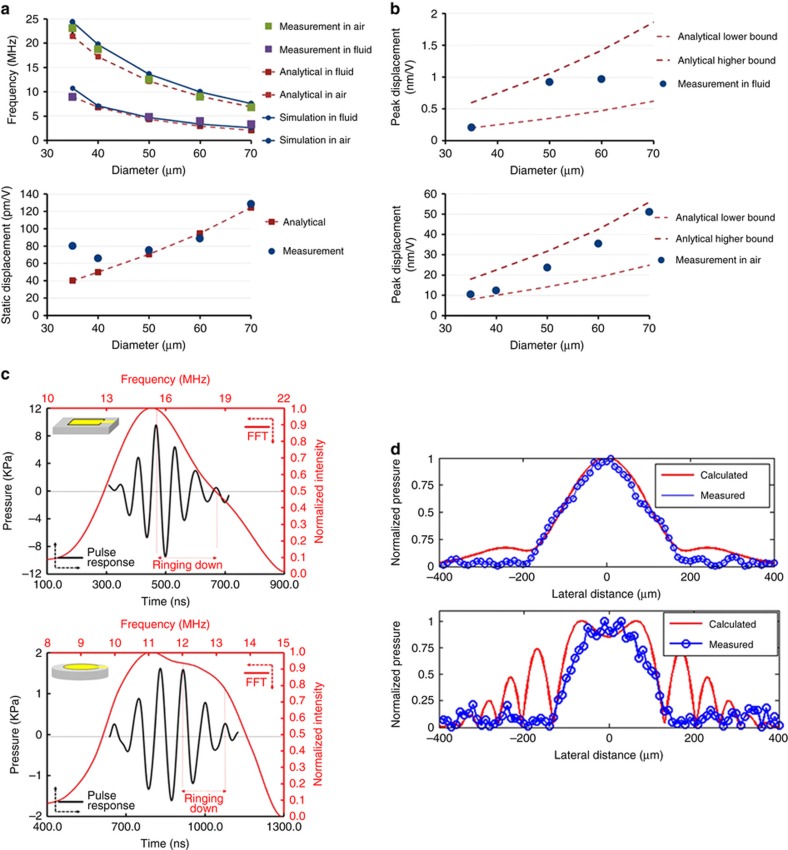
(**a**) Modeled and measured resonance frequency in air and fluid (top). Modeled and measured static displacement (bottom). (**b**) Modeled and measured peak displacement in fluid (top) and in air (bottom) (**c**) Pressure output of the rectangular (top) and circular (bottom) PMUT arrays measured with a hydrophone 220 and 400 μm from the piezoelectric micromachined ultrasonic transducer (PMUT) array. Black: time-domain; Red: FFT. (**d**) The normalized pressure field at a 500-μm distance from the PMUT array measured by translating the hydrophone along the y-axis when transmitting with one column (top) and three columns (bottom).

Circular PMUTs with five different diameters ranging from 35 to 70 μm were fabricated and measured in air and fluid (Fluorinert FC-70, 3 M) using a laser doppler vibrometer (LDV, OFV-5000; Polytec, Inc., Campbell CA, USA). The measured frequencies in air and fluid are in good agreement with the predictions from models, where *f*_0,air_ scales as 1/*l*^2^, as shown in Figure 5a. The measured displacement at resonance in air, when divided by the measured quality factor, yields a normalized displacement that can be compared with the static displacement *d*_s_ calculated from the analytical solution in Figure 5a. However, the static displacement *d*_s_ for a 35 μm PMUT is two times higher than the predicted value. As shown in Figure 5b, the measured peak displacement *d*_p_ for circular PMUTs with five different diameters is well predicted by the product of the static displacement *d*_s_ and the estimated quality factor. The measured displacement *d*_p_ scales as *l*^2^, in good agreement with the model in Equation (10). To quantify the die-to-die variability, five chips selected from locations across a 200 mm wafer were characterized, and the frequency and peak displacement response in air were recorded. The die-to-die variation in resonant frequency was small, varying by ~4%. However, the displacement variation was much greater, with an ~20% die-to-die difference in the peak amplitude observed for 50 μm PMUTs. Some of the amplitude variation may be measurement error due to imperfect placement of the LDV laser spot. Cross-section SEM images revealed the Si elastic layer of the PMUT varied by ~10% from die to die. Based on Equation (8) and Equation (10), where *f*_0_∝*t* and *d*_p_∝1/*t*^4^, the maximum frequency difference due to Si thickness variation is calculated to be 5%, while the amplitude variation is 30%. As a result, the frequency and peak displacement variations can be mostly attributed to Si layer thickness variation.

Acoustic tests were conducted with the PMUT array immersed in fluid with a 0.04-mm-diameter needle hydrophone (Precision Acoustics, Inc.) used to measure the pressure output. As shown in Figure 1d., the 30 μm×40 μm rectangular PMUTs were on a 43 μm×58 μm grid, while the 35 μm diameter circular PMUTs were on a 70 μm×80 μm grid. Driving a single column of PMUTs with two 24 V cycles at 14 MHz resulted in 9.4 kPa for the rectangular design measured with the hydrophone 220 μm away from the PMUT chip and 1.62 kPa for the circular design measured with the hydrophone at a 400 μm distance, as shown in Figure 5c. These pressure measurements correspond to acoustic surface pressures of 70.8 and 41.9 kPa for the rectangular and circular designs, respectively. This 1.7 factor of difference in the surface pressure agrees with the calculation from Equation (4), as the two PMUT designs have similar amplitude responses but differ primarily in that the rectangular design has a 3× higher fill-factor. The measured pressures are also in good agreement with the modeled pressure calculated from the measured displacement and frequency. The pressure variation due to die-to-die differences is estimated to be <50%, while the pressure was measured to vary by only 20% when the hydrophone distance was changed by 200 μm, both of which are relatively small compared to the measured difference in output pressure of the two designs. The beam width was measured by laterally translating the hydrophone at a distance of 500 μm away from the PMUT chip. The measured pressure profile, shown in Figure 5d, shows that the beam-width is 200 and 150 μm when transmitting with one column and three columns, respectively. The measured beam-width is in reasonable agreement with the analytical calculation from Equation (14), with differences resulting from the physical size of the hydrophone needle and errors in the tilt and positioning of the array.

**Figure 6:**
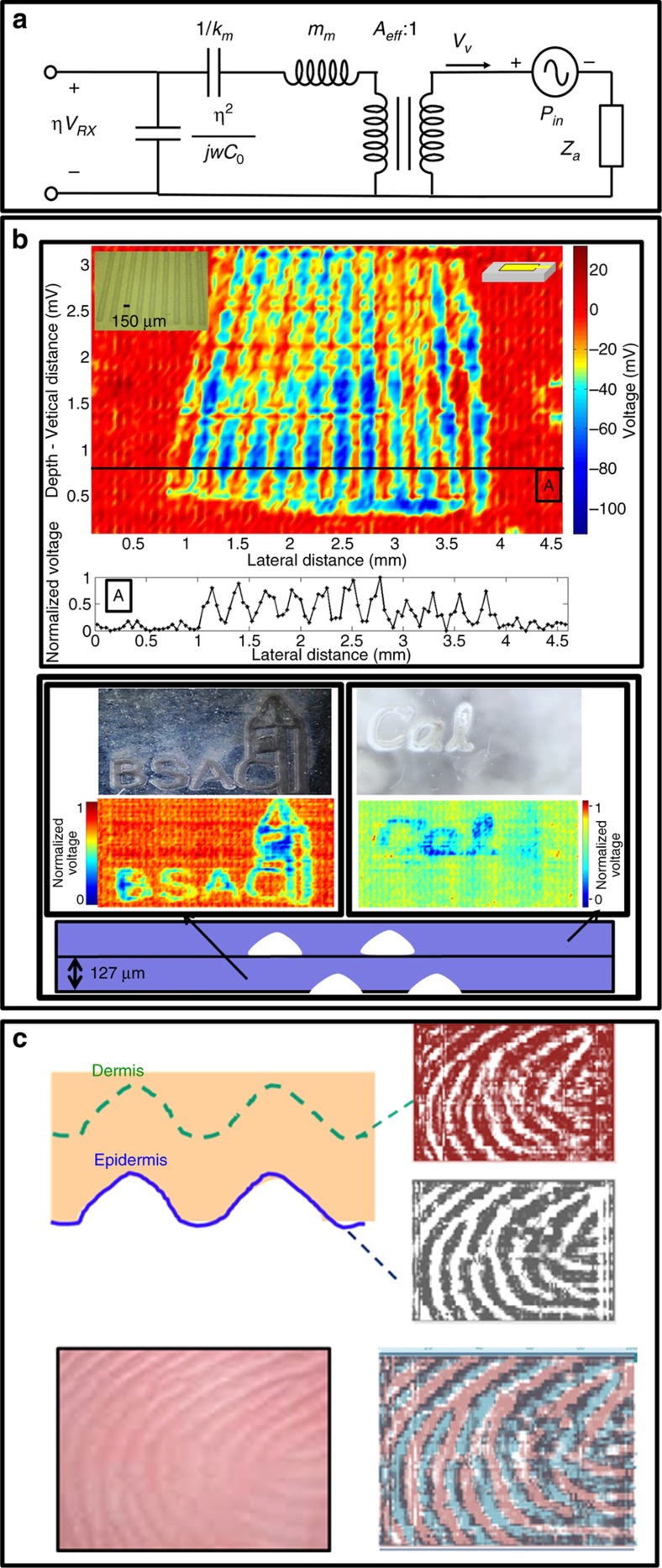
(**a**) Equivalent circuit model for receiving. (**b**) Pulse-echo images. Top: National Institute of Standards and Technology fingerprint resolution test composed of converging parallel lines. Bottom: axial resolution demonstration using two overlaid patterns separated by 127 μm in depth. (**c**) Ultrasound fingerprint image collected at the epidermis and sub-epidermis layer (top). Good agreement is observed between the ultrasonic images and the optical fingerprint image.

The rectangular PMUT array demonstrated to have the best performance was further characterized to understand its sensitivity, electromechanical coupling coefficient *k*_t_^2^ and insertion loss. Based on the equivalent circuit model in Figure 6a, the receiving sensitivity *S*_RX_ is

(16)SRX=VRXPRX=GAeffηη2Zeleη2Zele+Ztot

where *V*_RX_ and *P*_RX_ are the received voltage and pressure on the PMUT surface, respectively, *G* is the gain of the front-end amplifier, *Z*_ele_ is the electrical impedance of the PMUT and *Z*_tot_ is the sum of the mechanical and acoustic impedances of the PMUT. Pulse-echo experiments were conducted with the chip packaged with a 250 μm-thick layer of PDMS and an imaging phantom placed on top of the PDMS layer. Using the acoustic pressure output measured with the hydrophone together with the received signal amplitude from pulse-echo measurements, the pressure sensitivity was determined to be 2 mV kPa^−1^, which agrees with the sensitivity computed from Equation (16). The electromechanical coupling coefficient *k*_t_^2^ is estimated to be 0.3% from^16^

(17)kt2≅π2η28kmC0

where *η*=*kd*_s_ is the electromechanical coupling, while *k*_m_ and *C*_0_ are the mechanical stiffness and electrical capacitance of the PMUT, respectively. Considering the partial electrode coverage of the AlN thin film and that the AlN thickness is only 40% of the entire device thickness, the calculated *k*_t_^2^ is consistent with the value calculated from Refs. 11, 20 for an AlN piezoelectric thin film (1.49%). The array insertion loss was measured by exciting a column of PMUTs with a two-cycle 24 V_pp_ 14 MHz pulse and measuring the voltage from the reflected echo. The measured insertion loss is 90 dB from the transmit voltage and receive voltage, which includes approximately 8 dB of the spreading and absorption loss over the 500 μm round-trip path. The absorption coefficient of PDMS is 16.1 dB cm^−1^ or 0.8 dB for the 500 μm round-trip, much less than the spreading loss.

Axial and transverse image resolution experiments were conducted using two different phantoms. In each experiment, pulse-echo measurements are collected from each of the 110 columns in sequence, with a complete image formed in 2.6 ms. In each column’s TX cycle, five adjacent columns (*N*−2, *N*−1, *N*, *N*+1, *N*+2) of 56 PMUTs are excited without beamforming, and the center column (*N*) is used as receivers. Figure 6b shows a 2D pulse-echo ultrasonic image of a fingerprint sensor resolution test pattern fabricated by the National Institute of Standards and Technology (NIST; Figure 6b), demonstrating a 5:1 contrast ratio over the 4.6 mm×3.2 mm ultrasound image. A separate test characterized the lateral resolution to be 80 μm, consistent with the 80 μm beam width computed using the acoustic model. The discontinuous image pattern is due to a non-flat PDMS surface. To demonstrate the axial resolution, a phantom was constructed consisting of two overlaid patterns separated axially by 127 μm. Time-gated images collected at these two imaging depths clearly show the two patterns (Figure 6b). Similarly, human skin is composed of several layers, and ultrasonic images can be collected at the finger surface and at the dermal layer beneath the finger surface. Two time-gated fingerprint images collected at two depths are shown in Figure 6c. The sub-surface image matches the negative of the surface image. The two collected fingerprint images match the anatomy from Ref. 21. The characteristics of the 110×56 rectangular PMUT array are summarized in Table 1.

## Conclusions

This paper presented a single-chip ultrasonic fingerprint sensor that meets the resolution requirements for consumer electronics applications. A high fill-factor array of rectangular PMUTs was shown to achieve the best acoustic performance. The array produces an output pressure of 15 kPa at 240 V input to five columns of 56 PMUTs. The fractional bandwidth is 37%, sufficient to resolve images separated by an axial distance of 127 μm. Further optimization of the PMUT design may improve the fractional bandwidth to enable higher axial resolution.

## Figures and Tables

**Table 1:** Ultrasonic fingerprint sensor characteristics.

Center frequency *f*_0_	14 MHz
Pressure at the imaging plane (5 column TX Drive)	15 kPa
Receive sensitivity	2 mV kPa^−1^
Electromechanical coupling coefficient $k_{t}^{2}$	0.3%
Insertion loss	90 dB
Number of pixels	110×56
Pixel size	43×58 μm
Lateral/axial image resolution	75/150 μm
Contrast ratio	5:1
